# Clinical Scoring for Diagnosis of Acute Lower Abdominal Pain in Female of Reproductive Age

**DOI:** 10.1155/2013/730167

**Published:** 2013-12-14

**Authors:** Kijja Jearwattanakanok, Sirikan Yamada, Watcharin Suntornlimsiri, Waratsuda Smuthtai, Jayanton Patumanond

**Affiliations:** ^1^Department of Surgery, Nakornping Hospital, Chiang Mai 50180, Thailand; ^2^Division of Gastrointestinal Surgery and Endnoscopy, Department of Surgery, Faculty of Medicine, Chiang Mai University, Chiang Mai 50200, Thailand; ^3^Department of Obstetrics & Gynecology, Nakornping Hospital, Chiang Mai 50180, Thailand; ^4^Department of Emergency Medicine, Nakornping Hospital, Chiang Mai 50180, Thailand; ^5^Clinical Epidemiology Unit & Clinical Research Center, Faculty of Medicine, Thammasat University, Pathum Thani 12120, Thailand

## Abstract

*Background*. Obstetrics and gynecological conditions (OB-GYNc) are difficult to be differentiated from appendicitis in young adult females presenting with acute lower abdominal pain. Timely and correct diagnosis is clinically challenging. *Method*. A retrospective data analysis was performed on 542 female patients who were admitted to a tertiary care hospital with a chief complaint of acute lower abdominal pain. Diagnostic indicators of appendicitis and OB-GYNc were identified by stepwise multivariable polytomous logistic regression. Diagnostic performances of the scores were tested. *Result*. The developed clinical score is comprised of (1) guarding or rebound tenderness, (2) pregnancy, (3) sites of abdominal tenderness, (4) leukocytosis, (5) peripheral neutrophils ≥75%, and (6) presence of diarrhea. For diagnosis of appendicitis, the area under the ROC curve was 0.8696, and the sensitivity and specificity were 89.25% and 70.00%. For OB-GYNc, the corresponding values were 0.8450, 66.67%, and 94.85%, respectively. *Conclusion*. The clinical scoring system can differentiate the diagnosis of acute lower abdominal pain in young adult females. Time spent for diagnosis at the emergency room may be shortened, and the patients would be admitted to the appropriate departments in less time.

## 1. Introduction

Diagnosis of acute lower abdominal pain in young adult female is a clinical challenge. Appendicitis and obstetrics and gynecological conditions (OB-GYNc), such as ectopic pregnancy, pelvic inflammatory diseases, and complicated ovarian cyst, are common causes of acute lower abdominal pain in females during reproductive age [1]. Accurate and timely diagnosis of the condition is critical since incorrect diagnosis can lead to improper surgical intervention, and delayed diagnosis results in delayed management of urgent conditions [2].

Emergency physicians play an important role in early diagnosis and prompt management of the conditions. Experienced emergency physicians can detect important clinical findings and give a provisional diagnosis to a patient before transferring her to general surgery or obstetrics and gynecology departments according to their judgment. Previous studies showed that some clinical indicators were helpful to distinguish appendicitis and common obstetrics and gynecological conditions (OB-GYNc) from nonspecific abdominal pain (NSAP) [3].

To resolve the difficulty in diagnosis of acute lower abdominal pain in female patients, whose appendicitis is confounded by OB-GYNc, imaging studies had been done. Imaging investigations such as ultrasonography, computerized tomography (CT), and magnetic resonance imaging (MRI) have high accuracies in diagnosis of acute lower abdominal pain [4, 5]. However, the universal usage of CT may not be cost-effective in countries with limited healthcare resources [6]. In addition, time spent for such investigations is also important for the emergency department.

Clinical diagnostic scoring, on the other hand, may be more appropriate for early diagnosis in an emergency department setting. Clinical scoring for diagnosis of appendicitis was studied for its application as a guideline used for admission and investigations [7, 8]. However, such clinical scoring system was not designed for diagnosis of acute lower abdominal pain from obstetrics and gynecology conditions (OB-GYNc), which are also important in young adult females.

The objective of the present study was to develop a clinical scoring for diagnosis of acute lower abdominal pain in females of reproductive age that could either have appendicitis, OB-GYNc, or NSAP.

## 2. Method

### 2.1. Patients

We studied medical records of women aging between 15 and 50 years who were admitted to a surgical or obstetrics and gynecology department of a university affiliated tertiary care hospital, with a chief complaint of acute lower abdominal pain within 14 days during January–December 2008. Patients were categorized into 3 groups by their final diagnoses upon discharge. The first group was appendicitis (ICD-10 code K-35), the second group was obstetrics and gynecological conditions (OB-GYNc), such as ectopic pregnancy (ICD-10 code O-00), pelvic inflammatory disease (ICD-10 code N70), and complicated ovarian cyst (ICD-10 code N83). The third group was nonappendicitis and non-OB-GYNc (A-09 and R-10 or other causes of abdominal pain) which was classified as nonspecific abdominal pain (NSAP). The diagnostic criteria for appendicitis were the presentation of any gross inflammation of appendix in operative records or successful conservative treatment with antibiotics in appendiceal abscesses. All medical records were reviewed for operative records, pathological reports, imaging studies, and follow-up records to ascertain their final diagnoses.

### 2.2. Study Variables

Patients' characteristic (age and marital status), characteristics of pain and associated symptoms (duration of abdominal pain, shifting of pain location, and the presence of anorexia, nausea, vomiting, and diarrhea), and the presence of pregnancy and abnormal vaginal bleeding were reviewed. Body temperature, systolic blood pressure, location of abdominal tenderness, and presence of guarding or rebound tenderness on the first admission day were recorded. Initial laboratory results of complete blood count (hematocrit, white blood cell count, and percentage of neutrophil) and urine pregnancy test were noted. All these clinical indicators were studied for their predictive potential of the final diagnoses.

### 2.3. Missing Data Management

We had an assumption that the pattern of missing data was missing at random (MAR). Therefore, the multiple imputation method was used in data analysis. We imputed missing data 20 times using the multivariate normal regression method. The imputation model variables included all nonmissing variables and outcome variables (final diagnoses).

### 2.4. Data Analysis

#### 2.4.1. Derivation of Clinical Scoring

The predictive model for prediction of final diagnosis of appendicitis or OB-GYNc was derived from manual backward stepwise polytomous logistic regression with multiple imputation estimation method, by using NSAP as the base outcome. Nonsignificant clinical diagnostic indicators were manually removed from the model until the remaining coefficients were significant at *P* values less than 0.05 in one or both diagnoses. Item scores for appendicitis and OB-GYNc were derived from polytomous logistic coefficients of the corresponding diagnosis. We compared the sum of item scores for each diagnosis as the representative of diagnostic possibilities and designed an algorithm for prediction of diagnosis by the scoring system.

#### 2.4.2. Test for Score Performance

Performance of the scoring system was tested with the complete data set. Areas under the receiver operating characteristic (ROC) curves were calculated from disease-specific logistic models to determine discrimination abilities of the score. Accuracy of the scoring system was tested by comparing diagnosis suggested (predicted) from the scoring system with the final (true) diagnosis of patients, and diagnostic indices were calculated.

#### 2.4.3. Ethics

This study was approved from the Ethical Committee of the Faculty of Medicine of Chiang Mai University and the Ethical Committee of Nakornping Hospital.

## 3. Result

### 3.1. Patient Characteristic and Score Derivation

A total of 542 female patients were studied, of which final diagnosis were appendicitis in 382 patients, OB-GYNc in 97 patients, and NSAP in 63 patients. Of the OB-GYNc, 48 were diagnosed with ectopic pregnancy, 42 were complicated ovarian cysts, and 7 were pelvic inflammatory disease. The final diagnoses of NSAP were: abdominal pain without specific diagnosis (*n* = 31), enteritis/gastroenteritis (*n* = 21), diverticulitis (*n* = 5), urinary tract infection (*n* = 2), radiation enteritis (*n* = 2), ileitis (*n* = 1), and twisted omentum (*n* = 1). There were 453 patients who underwent surgery, 362 of appendicitis, 69 of OB-GYNc, and 22 of NSAP. Twenty of appendiceal abscesses were treated with antibiotics without surgery. Clinical diagnostic indicators with missing data were: pulse rate (1.6%), systolic blood pressure (1.8%), hematocrit (12.9%), white blood cell count (23.2%), and percentage of neutrophil (26.0%). Significant differences between diagnosis groups were seen in diagnostic indicators of shifting of pain, anorexia, nausea and vomiting, diarrhea, abnormal vaginal bleeding, body temperature, pulse rate, systolic blood pressure, site of abdominal tenderness, guarding or rebound tenderness, hematocrit, white blood cell count, percentage of neutrophil, and pregnancy (Table 1).

With multivariable analysis, significant clinical indicators were guarding or rebound tenderness, right lower quadrant (RLQ) tenderness, pregnancy, left lower quadrant (LLQ) tenderness, presence of diarrhea, and leukocytosis (defined as white blood cell ≥10,000/*μ*L). The item score of each clinical indicator for diagnosis of appendicitis or OB-GYNc derived from polytomous logistic coefficients (Table 2). Item scores for diagnosis of appendicitis (appendicitis score) were 1.8 for the presence of guarding or rebound tenderness, −1.7 for pregnancy, 1.5 for leukocytosis, 1.3 for neutrophil ≥75%, 1.5 for RLQ tenderness, 0 for LLQ tenderness, −1.4 for presence of diarrhea, and −1.5 for a constant. Item scores for the diagnosis of OB-GYNc (OB-GYN score) were 0 for the presence of guarding or rebound tenderness, 2.4 for pregnancy, 0 for leukocytosis, 1.6 for neutrophil ≥75%, 0 for RLQ tenderness, 1.9 for LLQ tenderness, and −2.3 for presence of diarrhea.

### 3.2. Performance of the Scoring System

**Figure 1:**
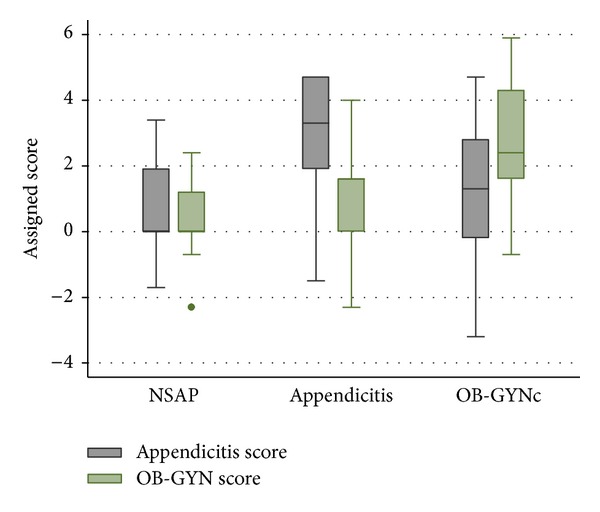
Distribution (box plot) of appendicitis score and OB-GYN score in nonspecific abdominal pain (NSAP), appendicitis, and common obstetrics and gynecological conditions (OB-GYNc).

The median (p25 and p75) of appendicitis score for diagnosis of NSAP was 0 (0, 1.9) for diagnosis of appendicitis was 3.3 (1.9, 4.7), and for diagnosis of (−0.2, 1.8) OB-GYNc was 1.3. The median (p25 and p75) of OB-GYNc score for NSAP was 0 (0, 1.2), 1.6 (0, 1.6) for appendicitis, and 2.4 (1.6, 4.3) for the diagnosis of OB-GYNc (Figure 1). Areas under ROC curves, which reflected discriminative abilities of appendicitis score and OB-GYN score, were 0.8696 for appendicitis versus NSAP and 0.8450 for OB-GYNc versus NSAP, respectively.

By the concept of relative probabilities, an algorithm for diagnosis from the scoring system was created (Table 3). When using this algorithm in 399 patients of the complete data set, the scoring system yielded correct diagnosis (comparing to final diagnosis) of appendicitis in 249 of 285 (positive predictive value, PPV, 87.37%) and correct diagnosis of OB-GYNc in 46 of 63 (PPV 73.02%) (Table 4).

The scoring system had a sensitivity of 89.25%, a specificity of 70.00%, and a likelihood ratio of positive test of 2.97 in diagnosis of appendicitis. For diagnosis of OB-GYNc, the scoring system had a sensitivity of 66.67%, a specificity of 94.85%, and a likelihood ratio of positive test of 12.94 (Table 5).

**Figure 2:**
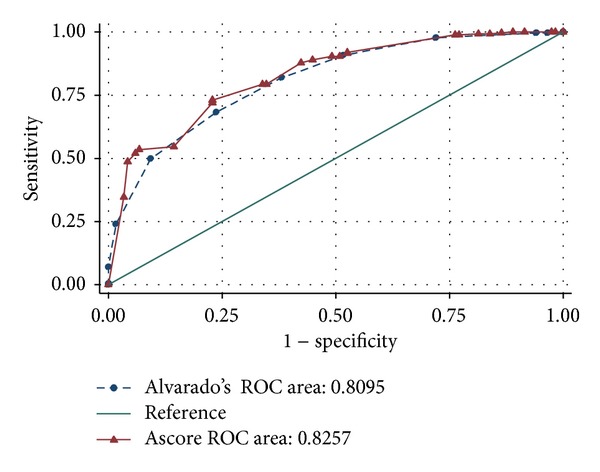
Receiver operating characteristic (ROC) curves of Alvarado's score (dash line) and appendicitis score (ascore, solid line) for diagnosis of appendicitis.

Alvarado's score was also calculated for each patient to compare with our scoring system. The means (±sd) of Alvarado's score were 6.19 ± 1.77 for appendicitis, 3.94 ± 2.06 for OB-GYNc, and 3.78 ± 1.63 for NSAP, respectively. At the “cut-off” point at 7, Alvarado's score yielded a sensitivity of 49.8%, a specificity of 90.7%, and a likelihood ratio of positive test of 5.34 for diagnosis of appendicitis. When comparing our appendicitis score and Alvarado's score in their abilities to discriminate appendicitis and “nonappendicitis,” the area under ROC of our appendicitis score was 0.8257 (95% CI: 0.78236, 0.86900) and the area under ROC of Alvarado's score was 0.8095 (95% CI: 0.76460, 0.85441). The two areas under ROC were not significantly different at a *P*-value of 0.270 (Figure 2).

## 4. Discussion

Acute lower abdominal pain in young adult females is a diagnostic challenge for general surgeons, gynecologists, and emergency physicians. Although ultrasonography and CT scan can increase diagnostic accuracy [2, 9, 10], evaluation of patients by clinical specialists is still needed. Diagnosis of acute lower abdominal pain in female patients is more difficult than in male patients; this reflects in negative appendectomies among females were observed more often [11–13]. In case of appendicitis, this can be explained by a wide range of clinical features of the disease [14].

The combination of clinical features and laboratory tests or clinical indicators is useful for the diagnosis of patients' conditions. Mathematically, these clinical indicators can be assigned as scores for diagnosis of difficult conditions. Alvarado's score, for example, was studied for the diagnosis of appendicitis in patients with abdominal pain with good results [15–17]. However, for young adult females, the diagnosis of obstetrics and gynecology conditions is also clinically important.

Polytomous logistic regression had been studied for the diagnosis of conditions that can have more than two possibilities [18, 19]. It can be applied in acute lower abdominal pain in women which could be either appendicitis, OB-GYNc, or NSAP. The present scoring system, which comprised of appendicitis and OB-GYN scores, was derived by applying polytomous logistic regression concept in comparing the relative probabilities of these two conditions. Note that the item scores contained positive and negative values, which reflected increase or decrease probabilities of the corresponding diagnoses in presenting of such clinical indicators.

If we use the present scoring system for diagnosis of acute lower abdominal pain in women of reproductive age, there would be “overdiagnosis” of appendicitis in 36 of 285 patients (4.91% were actually OB-GYNc, and 7.72% were NSAP) while “underdiagnosis” of appendicitis would be observed in 30 of 279 patients. However, a caution should be made to be aware of the scoring system diagnosis of NSAP because of the high risk for appendicitis and OB-GYNc. These patients should be subjected to close observation or further investigations.

Ultrasonography was also performed in uncertain cases in our institute. The results of ultrasonography were helpful in some cases, especially for diagnoses of OB-GYNc. There were 139 patients who underwent ultrasound in this study. Final diagnoses of OB-GYNc correlated well with ultrasound results (27/32 of ectopic pregnancies, 2/2 of PID, and 20/24 of complicated ovarian cysts). However, only 28 of 48 appendicitis patients were correctly diagnosed by ultrasound.

The problem of appendicitis in pregnancy was one caution in using the present scoring system. Of the 37 pregnant women with acute lower abdominal pain in the complete data set, all were categorized into OB-GYNc, which would be correct in 30 of them. However, 4 of them were appendicitis and 3 were NSAP. Therefore, investigation such as ultrasound might be of value for pregnant women with acute lower abdominal pain. Appendicitis should be suspected in pregnant women with right iliac fossa pain unless other causes of pain are evident.

The present scoring system has an advantage of high specificity and high negative predictive value in the diagnosis of OB-GYNc. This could help for ruling out OB-GYNc without further consultation with gynecologists, which may save some extra time in the emergency department. One disadvantage of the scoring system is the need to doubly compute both appendicitis score and OB-GYN score for comparison. However, an electronic calculator can be designed for such purpose.

Some limitations in this study should be taken into consideration. The incompleteness of lower abdominal pain patients was likely, because patients from the internal medicine department were not included. As our routine practice, the emergency department staff would transfer a young adult female with acute lower abdominal pain to either surgical department or obstetrics and gynecology department rather than to the department of internal medicine or other departments. This preselection may limit the present results to be generalized to other emergency departments. In normal practice, it would be difficult to preselect some patients with overlapping symptoms of abdominal pain. This might possibly explain the low number of patients with diverticulitis or other nonsurgical conditions in our study.

It may also be difficult to generalize the present scoring system to other settings, as it was derived from a tertiary care hospital. The clinical signs and symptoms of early presenting cases at primary settings may be quite different. The retrospective nature of the study may also limit its generalization. A prospective evaluation of the score in different settings should be conducted before it is used in routine clinical practice.

## 5. Conclusion

The present clinical scoring system can help clinicians distinguish appendicitis and OB-GYNc from NSAP in child-bearing age women with acute lower abdominal pain. It may be used as a guideline for admitting patients to the general surgery or the obstetrics and gynecology wards or requesting further investigations. However, validation of the scoring system is needed before being used in clinical practice.

## Figures and Tables

**Table 1:** Demographic characteristics and clinical findings of patients with appendicitis, obstetrics-gynecological conditions (OB-GYNc), and nonspecific abdominal pain (NSAP).

Characteristics	Appendicitis (*n* = 382)		OB-GYNc (*n* = 97)		NSAP (*n* = 63)		*P* value
	*n*	%	*n*	%	*n*	%	
Age (yr)							
15–20	106	27.8	16	16.5	16	25.4	
21–30	107	28.0	44	45.4	18	28.6	
31–40	74	19.4	25	25.8	14	22.2	
41–50	95	24.9	12	12.4	15	23.8	
Mean (SD)	30.1	(11.3)	28.9	(8.8)	29.9	(10.4)	0.937*
Single	193	50.8	49	51.0	33	53.2	0.943
Duration of pain (hr)							
Mean (SD)	31.2	(32.0)	52.4	(65.9)	34.9	(37.4)	0.413*
Shifting of pain	142	31.2	6	6.2	11	17.5	<0.001
Anorexia	43	11.3	2	2.1	6	9.5	0.010
Nausea and vomiting	200	52.4	15	15.5	20	31.8	<0.001
Abnormal vaginal bleeding	1	0.1	28	28.9	2	3.2	<0.001
Diarrhea	29	7.6	4	4.1	13	20.6	0.002
Temperature ≥ 37.5°C	124	33.3	14	14.6	12	19.4	<0.001
Pulse rate (/min)	(*n* = 374)		(*n* = 97)		(*n* = 62)		
Mean (SD)	90.8	(15.5)	88.0	(17.4)	85.2	(17.0)	0.021
Systolic blood pressure (mmHg)	(*n* = 374)		(*n* = 97)		(*n* = 61)		
Mean (SD)	121.8	(15.9)	112.4	(18.5)	117.9	(14.3)	<0.001
RLQ tender	374	97.9	71	73.2	53	84.1	<0.001
LLQ tender	15	3.9	48	49.5	6	9.5	<0.001
Guarding/rebound tenderness	255	66.8	34	35.1	13	20.6	<0.001
Hematocrit (%)	(*n* = 336)		(*n* = 86)		(*n* = 55)		
Mean (SD)	38.0	(3.9)	33.3	(6.0)	36.5	(5.9)	<0.001*
WBC (/*μ*L)	(*n* = 292)		(*n* = 71)		(*n* = 53)		
Mean (SD)	14204.5	(4638.4)	11875.9	(4531.9)	9958.8	(5200.0)	<0.001*
Neutrophil (%)	(*n* = 281)		(*n* = 69)		(*n* = 51)		
≥75	171	60.9	39	56.5	10	19.6	<0.001
Pregnant/positive pregnancy test	7	1.8	47	48.5	3	4.8	<0.001

*Kruskal-Wallis equality-of-populations rank test, SD: standard deviation, RLQ: right lower quadrant, and LLQ: left lower quadrant.

**Table 2:** Coefficients (95% confidence interval: CI) and assigned item scores of selected predictors for diagnosis of appendicitis or common obstetrics and gynecological conditions (OB-GYNc), from polynomial logistic regression analysis*.

Predictors	Appendicitis coefficients (95% CI)	*P* value	OB-GYNc coefficients (95% CI)	*P* value	Assigned score	
					Appendicitis score	OB-GYN score
Guarding/rebound tenderness	1.85 (1.12, 2.59)	<0.001	0.40 (−0.54, 1.34)	0.407	1.9	0
Pregnancy	−1.70 (−3.28, −0.12)	0.035	2.39 (1.05, 3.73)	<0.001	−1.7	2.4
Leukocytosis	1.53 (0.78, 2.29)	<0.001	−0.13 (−1.11, 0.84)	0.787	1.5	0
Neutrophil ≥ 75%	1.25 (0.35, 2.15)	0.007	1.61 (0.49, 2.73)	0.005	1.3	1.6
RLQ tenderness	1.52 (0.40, 2.64)	0.008	−0.42 (−1.46, 0.62)	0.429	1.5	0
LLQ tenderness	−1.11 (−2.26, 0.05)	0.062	1.93 (0.87, 2.98)	<0.001	0	1.9
Diarrhea	−1.44 (−2.41, −0.48)	0.003	−2.26 (−3.79, −0.74)	0.004	−1.4	−2.3
Constant	−1.45 (−2.61, −0.30)	0.014	−0.57 (−1.63, 0.49)	0.290	−1.5	0

*NSAP as baseline group, RLQ: right lower quadrant, and LLQ: left lower quadrant.

**Table 3:** Criteria for diagnostic preferences in acute lower abdominal pain, using the derived scores.

Diagnostic preferences	Criteria
Appendicitis	Appendicitis score > OB-GYN score and appendicitis score > 0
Common OB-GYN conditions (OB-GYNc)	OB-GYN score ≥ appendicitis score and OB-GYN score > 0
Nonspecific abdominal pain (NSAP)	Appendicitis score ≤ 0 and OB-GYN score ≤ 0

**Table 4:** Performance of the diagnostic preferences using the derived scoring scheme for appendicitis, common obstetrics and gynecological conditions (OB-GYNc), and nonspecific abdominal pain (NSAP).

Preference diagnosis using the scoring scheme	Final (true) diagnosis			Total
	Appendicitis	OB-GYNc	NSAP	
Appendicitis (%)	249 (87.37)	14 (4.91)	22 (7.72)	285 (100)
OB-GYNc (%)	9 (14.29)	46 (73.02)	8 (12.70)	63 (100)
NSAP (%)	21 (41.18)	9 (17.65)	21 (41.18)	51 (100)

Total (%)	279 (69.92)	69 (17.29)	51 (12.78)	399 (100)

**Table 5:** Diagnostic indices (and 95% confidence interval; CI) of the scoring scheme for diagnosis of appendicitis and common obstetrics and gynecological conditions (OB-GYNc).

Diagnostic indices	Appendicitis (95% CI)	OB-GYNc (95% CI)
Sensitivity (%)	89.25 (85.01, 92.63)	66.67 (54.29, 77.56)
Specificity (%)	70.00 (60.96, 78.02)	94.85 (91.88, 96.97)
Positive likelihood ratio	2.97 (2.26, 3.92)	12.94 (7.91, 21.17)
Negative likelihood ratio	0.15 (0.11, 0.22)	0.35 (0.25, 0.49)
Positive predictive value (%)	87.37 (82.94, 90.99)	73.02 (60.35, 83.42)
Negative predictive value (%)	73.68 (64.61, 81.49)	93.15 (89.90, 95.61)
